# Leveraging the Power of Knowledge Management to Transform Global Health and Development

**DOI:** 10.9745/GHSP-D-14-00228

**Published:** 2015-04-27

**Authors:** Tara M Sullivan, Rupali J Limaye, Vanessa Mitchell, Margaret D’Adamo, Zachary Baquet

**Affiliations:** ^a^​ Johns Hopkins Center for Communication Programs, Baltimore, MD, USA; ^b^​ United States Agency for International Development (USAID), Bureau for Global Health, Office of Population and Reproductive Health, Washington, DC, USA; ^c^​ USAID Bureau for Food Security, Washington, DC, USA

## Abstract

Good knowledge is essential to prevent disease and improve health. Knowledge management (KM) provides a systematic process and tools to promote access to and use of knowledge among health and development practitioners to improve health and development outcomes. KM tools range from publications and resources (briefs, articles, job aids) and products and services (websites, eLearning courses, mobile applications), to training and events (workshops, webinars, meetings) and approaches and techniques (peer assists, coaching, after-action reviews, knowledge cafés).

By its very nature, global health and development work involves a multitude of actors working toward common goals that transcend geographic, sectoral, organizational, and financial boundaries. These efforts require immediate access to the latest research and know-how and demand optimal use of limited resources to achieve maximum impact.^1^ Knowledge management (KM) can improve coordination, enhance learning and knowledge application, and improve capacity, thus heightening service quality, strengthening health systems, and, ultimately, improving health and development outcomes.

The purpose of this article is to demystify KM and advocate its increased use in global health and development projects. We first describe knowledge, define KM, and highlight some KM tools. Then we trace the history of KM as used in the private sector and in health and development. Finally, we illustrate through a case study from Bangladesh how KM can be used to support health and development outcomes.

## WHAT DOES KM REALLY MEAN?

Knowledge is the capacity to act effectively.^2^ Few would argue this is essential in our fight to prevent disease and improve health. Knowledge, or know-how, draws from our experience and allows us to solve problems using a combination of information and contextual understanding.^4^

There are a variety of KM definitions in use. What is common to most definitions is that KM is a systematic or intentional process that is linked to a broader set of organizational or project objectives. For this paper, we define *knowledge management* as the systematic process of collecting and curating knowledge and connecting people to it so they can act effectively.^2^^,^^3^

Two types of knowledge are managed: explicit and tacit. *Explicit knowledge* is easy to express in words and can be shared in written documents, manuals, or databases.^4^ On the other hand, *tacit knowledge*—that is, knowledge that lives “in our heads”—is difficult to articulate and is best shared through discussion, stories, observation, and personal interaction.^5^ Knowledge management gives us the ability to tap into and share explicit and tacit knowledge and to translate that knowledge into action.

**FIGURE 1.**
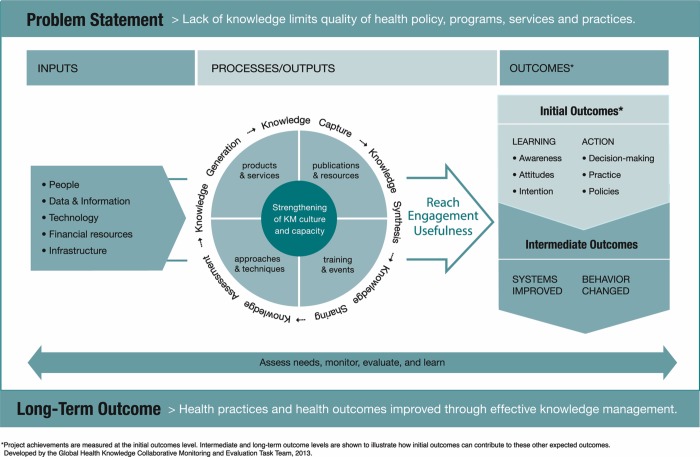
Knowledge Management for Global Health Logic Model

Within global health, lack of knowledge limits the quality of health policy, programs, services, and practices, but effective knowledge management can improve the situation. Knowledge management can be applied to boost an organization’s efficiency and effectiveness, or it can be used to improve service delivery throughout a health system. The Knowledge Management for Global Health Logic Model shows how KM program inputs, processes, and outputs work together to achieve intended health outcomes (Figure 1). KM inputs (people, data and information, technology, financial resources, and infrastructure) feed into 5 processes that make up the knowledge cycle (knowledge assessment, generation, capture, synthesis, and sharing) that, in a myriad of combinations, creates KM outputs, or tools.^5^ Knowledge management processes are supported by a strong KM culture and strengthened KM capacity. KM tools are measured in terms of reach, engagement, and usefulness and result in learning and action. Drawing on Rogers’ Diffusion of Innovation theory of how people adopt a new idea,^6^ the logic model illustrates how people generally move through an “innovation-decision process” when putting new knowledge to use, from initial awareness of the knowledge and intention to use that knowledge (learning) to actually using the new knowledge through informed decisions, improved practices, and better policies (action). These actions translate into strengthened systems, changed behavior, and, ultimately, improved health outcomes.

KM is a systematic process of collecting and curating knowledge and connecting people to it so they can act effectively.

Since development of the Knowledge Management for Global Health Logic Model, KM practitioners have come to further appreciate the central role of human interaction and people in the transfer and uptake of knowledge and the key role that KM can play in coordinating complex global health work.

Knowledge management tools can be classified into 4 categories, involving a range of low- to higher-end technologies: publications and resources, products and services, training and events, and approaches and techniques (Box). Publications and resources, such as policy briefs, guidelines, journal articles, and job aids, can help ensure health professionals have the knowledge and skills they need to do their jobs. Products and services, many of which harness the latest digital and mobile technologies, such as electronic repositories of essential information (toolkits and websites), eLearning courses, and mobile applications, can help share information quickly and widely. In addition, KM approaches and techniques such as peer assists (bringing together a group of peers to elicit feedback on a problem, project, or activity and draw lessons from the participants' knowledge and experience), coaching, mentorship, storytelling, and online or face-to-face forums such as communities of practice, are KM tools that can assist in enhancing coordination, learning, and adaptation. Other KM tools include after-action reviews (a structured debrief of an activity to learn how it can be done better in the future) and knowledge cafés (a type of meeting structure, which aims to provide an open and creative conversation on a topic of mutual interest to surface participants’ collective knowledge, share ideas and insights, and gain a deeper understanding of the subject and the issues involved), as well as trainings and events such as workshops, seminars, meetings, and webinars.

**BOX. Knowledge Management Tools**

**Publications and Resources**

- Fact sheets
- Guidelines
- Handbooks
- Job aids
- Journal articles
- Manuals
- Policy or research briefs
- Project reports

**Products and Services**

- Databases
- eLearning platforms
- Help desks
- Mobile applications
- Resource centers or libraries
- Websites and Web portals

**Training and Events**

- Conferences
- Forums
- Meetings
- Seminars
- Webinars
- Workshops

**Approaches and Techniques**

- After-action reviews
- Coaching
- Communities of practice
- Knowledge cafés
- Peer assists
- Study tours
- Twinning

## HOW HAS KM EVOLVED AS A DISCIPLINE?

Practical needs for knowledge expertise and understanding have been present for millennia.^7^ However, in the last 3 decades, primarily for-profit businesses have transformed KM into a discipline that focuses on people, processes, and technology to ensure the knowledge necessary for an organization to succeed is captured, created, synthesized, shared, and leveraged for maximum benefit.^8^

KM focuses on people, processes, and technology.

First-generation KM focused on technology, codification, and efficiency, with the goal of providing access to explicit knowledge.^9^ This technology- and codification-focused approach was intended to increase efficiency within mostly private-sector organizations^10^ ^–^ ^12^ but paid minimal attention to how or why knowledge is generated.^9^ The next generation focused on learning to maximize knowledge sharing, as technology applications that facilitated improved interaction became increasingly accessible.^13^ However, practitioners and scholars began to recognize that, despite technology’s ability within many companies to successfully leverage knowledge in many ways, it was not the only tool needed to deliver comprehensive knowledge management.^14^ The current generation uses people-focused approaches, such as after-action reviews, peer assists, and knowledge cafés. However, it has been noted that simply focusing on people and the technologies and tools they use does not automatically lead to knowledge application. Rather, the interaction between these factors is what allows an organization to manage knowledge effectively, and this application is dependent on a nurturing environment, including capacity building.^15^

All these trends have pointed to the need to better incorporate the most critical factors of the world we live in—human and social factors. Social knowledge management (social KM) promises to be the next generation of KM, building upon past generations while adding what has been the missing piece—the power of social. *Social KM* is driven by *social benefit* and recognizes the importance of *social capital, social learning, social media,* and *social networks*, all within the context of a larger *social system*.

Social KM promises to better incorporate human and social factors into KM processes and tools.

## HOW HAS KM BEEN USED IN GLOBAL HEALTH AND DEVELOPMENT?

Application of KM to global health and development has evolved over time, and as a result, interest has been growing among health care professionals about the importance of capturing, sharing, and using explicit and tacit knowledge.^16^ Initially, development organizations created centralized databases, such as the Development Experience Clearinghouse (DEC) from the United States Agency for International Development (USAID), and assumed users would find the database and content useful. Organizations also pushed explicit knowledge (for example, reports and data) to potential users, which—thanks to the expansion of the Web—was becoming easier and less expensive to do. However, limited attention was paid to how such knowledge would or could be used and even less attention was given to how people drove the flow of knowledge within groups, networks, and organizations.

The World Bank played a critical role in highlighting the strategic importance of knowledge sharing, after incorporating the idea of the “knowledge bank” as a central element of its work in the late 1990s.^17^ Knowledge capture, synthesis, analysis, and sharing require potential users to seek knowledge proactively and to adapt or modify it. Understanding this, development practitioners started to focus more on the ways people use expertise and less on the technology used to facilitate sharing. The Bank was one of the first to organize Knowledge Fairs, where thematic groups could display their knowledge sharing activities and could further illustrate with concrete examples the benefits of working together. The KM program director and staff used storytelling to sensitize the organization to the idea that sharing knowledge would enhance its organizational performance and, therefore, its global impact on poverty.

Many communities of practice formed around this time, and USAID, for example, supported the establishment of working groups, which provided a venue for the agency and its partners to collaborate informally around such issues as communication, monitoring and evaluation (M&E), social media, and even KM itself. It was during this time that USAID supported the development of online and face-to-face communities, including Microlinks (microfinance, 2004), FRAMEweb (environment, 2003), Agrilinks (agriculture and food security, 2011), the Virtual Leadership Development Program (management and leadership, 2002), and the Knowledge Gateway (global health, 2004).

Today KM continues to be an important discipline that global health and development organizations use to make their work more efficient and effective through its ability to transform health care delivery systems.^8^ Scholars have recognized that *information* is explicit and factual, while *knowledge* results from the integration of information with belief and context. This implies that while information can flow easily, knowledge is embedded in people and must be extracted to bridge the gap between knowledge and its application in policy and practice.^18^

Knowledge results from the integration of explicit information with belief and context.

Evaluations of global health-related KM suggest KM can help impact clinical practice, which, in turn, can improve health outcomes. For example, one study suggests that through eLearning, users are able to learn at their own pace, use customized training tools, and save both time and costs of travel to attend a class, increasing the potential for knowledge gain.^19^ Knowledge exchange portals create platforms for exchanging evidence-based information through online libraries, accessing epidemiological and demographic data, and creating or maintaining communities of practice.^20^ A randomized controlled trial reported that knowledge exchange portals, combined with tailored messaging services, can be effective at encouraging evidence-based policy and program design.^21^ A recent evaluation of a community of practice intended to build a critical mass of experts on performance-based financing by sharing expertise showed the community of practice had indeed become the central platform for knowledge sharing on this topic.^22^ In addition, KM tools and processes implemented at multiple levels of the health system in Malawi yielded improvements in knowledge exchange and health service delivery.^23^

Evidence on KM thus far has primarily focused on the impact of eLearning, portals, and platforms on health service delivery. The following case study from the Bangladesh Knowledge Management Initiative (BKMI) provides an example of how KM, particularly in the areas of learning and adaptation, coordination, and capacity strengthening, has been used to support health and development in Bangladesh.

## A KM CASE STUDY FROM BANGLADESH

Bangladesh has a population of more than 160 million people. In the last 20 years, child mortality has declined substantially (from 50 to 11 deaths per 1,000 live births between 1993/94 and 2011), total fertility has dropped (from 4.3 to 2.3 children per woman between 1991 and 2011), and use of modern contraception has increased (from 31% of currently married women in 1991 to 52% in 2011).^24^ Yet deaths in the first month of life now account for more than 60% of all under-5 deaths, and the nation has among the world’s highest malnutrition rates.^24^

Social and behavior change communication (SBCC) for health is an evidence- and theory-based process designed to improve health behavior and outcomes. Using communication strategies to change knowledge, attitudes, norms, and behavior within a particular socio-ecological context, SBCC practitioners recognize that the social and cultural environment can influence barriers to change—and action. The most effective SBCC programs are strategically designed and implemented so the selected mix of media approaches (for example, interpersonal, group, and mass media) results in maximum exposure to and mutually reinforcing messaging across all levels of the socio-ecological system.

**FIGURE 2.**
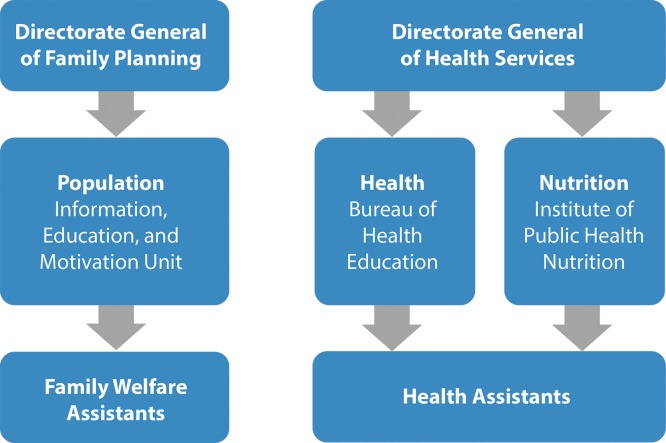
Bangladesh Ministry of Health and Family Welfare Structures Targeted by the Bangladesh Knowledge Management Initiative

Within the Bangladesh Ministry of Health and Family Welfare (MoHFW), 3 distinct government units design SBCC activities covering particular aspects of health (Figure 2):

- Health activities, including those focused on maternal and child health, are designed through the Bureau of Health Education (BHE) Unit of the Directorate General of Health Services
- Population activities, largely focused on family planning, are created through the Information, Education, and Motivation (IEM) Unit of the Directorate General of Family Planning
- Nutrition activities are carried out through the Institute of Public Health and Nutrition (IPHN) Unit of the Directorate General of Health Services

Two cadres of government field workers are primarily responsible for counseling clients at the clinic and household level: family welfare assistants (FWAs), who counsel and educate community members specifically about family planning, and health assistants (HAs), who counsel and communicate about general health issues such as maternal and child health, immunizations, and nutrition. As both cadres have distinct technical mandates, the field workers typically are not able to address health issues outside their scope that arise during counseling sessions.

In early 2011, the Knowledge for Health (K4Health) Project—the flagship project for KM in family planning funded by USAID’s Office of Population and Reproductive Health—was invited by USAID/Bangladesh and the MoHFW in Bangladesh to undertake a scoping visit to identify issues that could be resolved by applying KM solutions to their SBCC work.

The project found that within each ministry unit, capacity for SBCC was low, as was overall SBCC coordination among ministry staff. Furthermore, coordination of SBCC activities between ministry units and implementing partners was weak, leading to duplication of effort and a lack of awareness of the scope of SBCC programs in the country. At the field level, government field workers had little access to information and training, and they needed stronger counseling skills to communicate integrated health, population, and nutrition (HPN) messages to clients.

**FIGURE 3.**
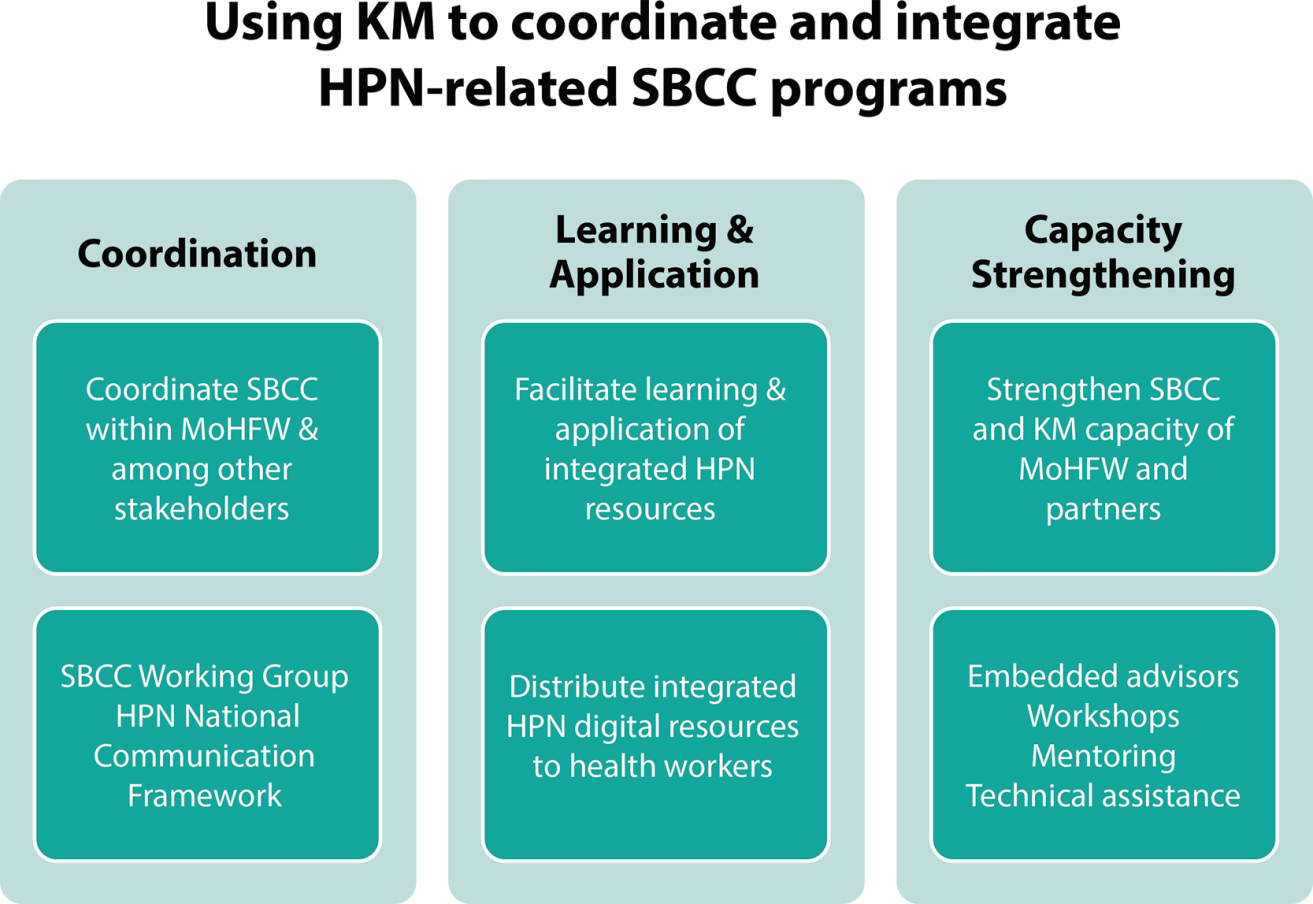
Key Components of the Bangladesh Knowledge Management Initiative. Abbreviations: HPN, health, population, and nutrition; KM, knowledge management; MoHFW, Ministry of Health and Family Welfare; SBCC, social and behavior change communication.

Subsequently, the Bangladesh Knowledge Management Initiative (BKMI) was implemented by K4Health from July 2011 to December 2013, focusing on 3 components to support SBCC (Figure 3):

1. Coordination of health, population, and nutrition SBCC programs and materials
2. Learning and application of an integrated package of health, population, and nutrition SBCC materials
3. Strengthening the SBCC capacity of the 3 units of the MoHFW

**FIGURE 4.**
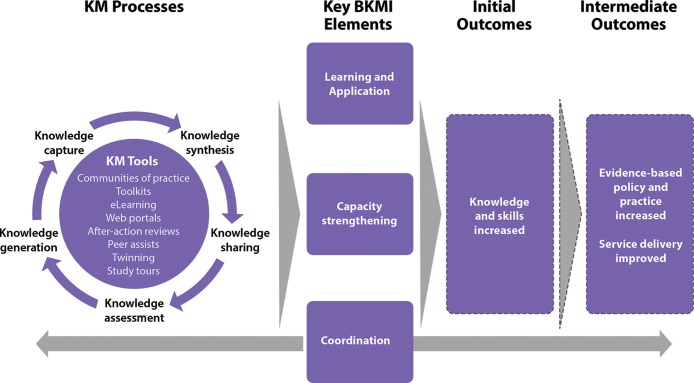
Conceptual Framework of the Bangladesh Knowledge Management Initiative

Building on the Knowledge Management for Global Health Logic Model, the BKMI conceptual framework theorized that KM processes and tools could work together to contribute to the 3 key components of the project of SBCC coordination, learning and application, and capacity strengthening (Figure 4). Coordination was an ongoing and integral part of the project that supported the learning and application and capacity strengthening components. Improvements in the 3 key project components were hypothesized to yield enhancements in SBCC knowledge and skills among MoHFW stakeholders and partner organizations as well as improvements in service quality.

### Coordination of Health, Population, and Nutrition SBCC Activities and Materials

The large SBCC community in Bangladesh, made up of government bodies, local and international NGOs, donor agencies, and private sector organizations, often worked in isolation from one another and was not always aligned with the MoHFW SBCC strategies. Similarly, the MoHFW was not fully aware of all the SBCC activities in the country, so there was a clear coordination gap. It was common to find several organizations working on SBCC projects with similar objectives, similar audiences, and in similar geographic areas as a result of failure to communicate and an absence of mechanisms for coordination. This duplication of effort can slow progress, lead to financial waste, and confuse the target audience with inconsistent messaging. There was a clear need for BKMI and the MoHFW to address inconsistent, outdated, and fragmented communication practices with cross-sectoral coordination and harmonization of HPN SBCC activities.

Lack of coordination between organizations led to duplication of effort.

The Bangladesh SBCC Working Group was established in March 2011, following a round-table discussion in which donors, the MoHFW, and NGOs identified the need for better coordination of HPN activities. When BKMI began working in Bangladesh in July 2011, it was selected to serve as the secretariat for the working group. Membership included the MoHFW, development partners, NGOs, and the private sector. This group met every 2 months to share knowledge and identify synergies among SBCC stakeholders working across HPN. Representatives from the SBCC community voluntarily and actively participated in the working group to learn from each other and to update each other on progress and upcoming events. BKMI supported the group by convening stakeholders, developing the agenda, and arranging logistics. BKMI also hosted a website to store and share key documents and created a listserv for the working group’s announcements.

Toward the end of the BKMI project, the SBCC Working Group was officially institutionalized, and the MoHFW took ownership of the group. A subgroup of the SBCC Working Group began mapping existing HPN communication programs and activities in the country to help ensure coordination. A second subgroup, called the Strategy Review Subgroup, conducted a review of 17 SBCC strategies and 4 operational plans with strong HPN communication components in Bangladesh to identify gaps, inconsistencies, synergies, and opportunities for cross-sector linkages. The last subgroup, called the Health, Population, and Nutrition SBCC Best Practices Subgroup, began to define, identify, and share HPN best practices.

In November 2012, the SBCC Working Group organized a 2-day workshop to align all HPN stakeholders and partners around a country-wide shared communication implementation framework and to develop a plan of action that coordinated activities across all partners in support of the MoHFW’s Health, Population and Nutrition Sector Development Program (HPN SDP), 2011–2016. Over the course of the 2 days, KM tools such as peer learning, small group work, and guided imagery with visual reporting were introduced. For example, small cross-sectoral groups were formed at roundtables to encourage peer-to-peer learning, and the workshop facilitator took participants through a guided imagery exercise as part of the visioning for the framework. Participants were tasked with imagining and then drawing what Bangladesh would look like with improved coordination and alignment of activities and SBCC messages. Participants then shared their drawings at their tables, and each table produced a common picture that was shared in a plenary. Common themes across the pictures were identified, which informed the vision for the framework.

Relevant stakeholders and experts continued to develop the framework in a participatory and iterative manner. Finalized in 2013, the framework is now being used to inform communication strategies, guide resource allocation, identify opportunities for collaboration, and guide implementation of SBCC activities.

These coordination activities, aimed at instituting high-quality standards and processes for the development of SBCC programs, were aligned with the strategic plan for the 2011–2016 HPNSDP.

### Learning and Application of Integrated HPN SBCC Activities and Materials

Because the FWAs and HAs addressed only a single set of issues during their respective counseling sessions (FWAs focused on family planning while HAs covered health and nutrition issues), they missed an opportunity to educate and counsel clients comprehensively about other important health issues. In the past, these field workers also struggled to do their jobs effectively given a number of other constraints. For example, they had little access to up-to-date information, few opportunities for continuing education, insufficient (or, conversely, overwhelming) counseling materials, and job aids that often contained conflicting communication messages. This resulted in confusion for both field workers and clients.

To address these challenges, BKMI conducted an eHealth pilot from May to August of 2013 in 2 districts (Chittagong and Sylhet) that have relatively high total fertility rates and low contraceptive prevalence rates, as well as poor maternal and child health and nutrition status, compared with other districts in the country. To improve field workers’ knowledge and skills in the use of information and communication technologies and their ability to integrate messages, including the ability to counsel on the full range of HPN topics, 300 field workers (150 FWAs and 150 HAs) received netbooks containing a digital HPN Toolkit and 8 video-based eLearning courses.

The Toolkit contained 116 HPN SBCC materials, including TV spots, flip charts, brochures, posters, and job aids, vetted by both the MoHFW and the field workers themselves. The MoHFW and subject matter experts used a standardized assessment tool (with 9 criteria) developed by the BKMI team that measured technical accuracy and quality of the materials and alignment with MoHFW priorities. The field workers then vetted the high-scoring materials with a separate assessment tool (also 9 criteria) for comprehension, appropriateness of messaging, and usability in counseling. (See supplementary materials for the assessment tool used by subject matter experts and the tool used by field workers.) The final materials that were vetted by both the subject matter experts and the field workers were uploaded into the Toolkit. The Toolkit is considered the gold-standard package of HPN SBCC resources for field workers in Bangladesh.

The eLearning package, designed to address the training needs of the field workers, included 2 family planning courses; 2 maternal, newborn, and child health courses; 2 nutrition courses; a course on interpersonal communication and counseling; and a course on integrated messaging. Courses contained 15- to 20-minute self-paced videos designed for low-literacy audiences. The project periodically assessed the field workers’ knowledge to measure learning from topics covered in the courses. These KM tools (digital Toolkits and eLearning courses) connected field workers to the knowledge and materials they needed to act effectively in their work. Preliminary results indicate the package enhanced field workers’ knowledge in family planning, exclusive breastfeeding, complementary feeding, and maternal health including danger signs.

### Strengthening KM and SBCC Capacity

From the outset, BMKI, in collaboration with the MoHFW, decided to second SBCC advisors within each of the 3 units to provide hands-on coaching and mentorship to staff. The units had limited capacity to coordinate activities across HPN, a problem that KM could solve through this secondment. To guide this work, BKMI conducted a 2-part baseline assessment in each of the government units to: (1) understand individual SBCC capacity needs, and (2) understand KM efforts currently being used to support SBCC within the unit. (See supplementary materials for the SBCC and KM capacity assessment tools.)

Specifically, the SBCC assessment tool measured capacity to:

- Conduct a situation analysis (including use of frameworks or models, research data to design SBCC programs, and activity reviews of other stakeholders to avoid duplication)
- Develop a communication strategy (including audience segmentation, communication objectives, and messaging)
- Develop materials (including creative briefs, concept testing, pretesting)
- Implement, manage, and lead programs (including work plan development, staffing plans and competencies, and supervision)
- Monitor, evaluate, and replan (including frameworks and mechanisms for measurement and the use of results for replanning)

The KM assessment measured each unit’s capacity to:

- Create and use KM processes in support of SBCC (including familiarity with concepts and the existence of systems for identifying and filling knowledge gaps and identifying tacit knowledge among staff)
- Manage and lead SBCC programs using KM (including development of strategies to disseminate and promote lessons learned and use of learning to strengthen existing skills among staff)
- Nurture support for KM (including providing forums for knowledge sharing, fostering staff responsibility for their own learning, and developing a strategy to deliver SBCC programs through digital platforms)
- Monitor, evaluate, and replan SBCC programs using KM (including use of frameworks, M&E data, and results to assess program progress and improve current programs)

Initially, all 3 units had low SBCC capacity and weak KM processes. Baseline data found SBCC programs did not use evidence-based design, SBCC materials and message development were of low quality, and M&E of SBCC activities was limited. In addition, standard processes for KM and SBCC were nearly non-existent.

To address the identified capacity building needs, BKMI, in collaboration with the unit line director and SBCC program managers, developed unit-specific capacity strengthening plans to improve SBCC skills and each unit’s overall culture to support KM. To strengthen individual SBCC capacity, BKMI arranged workshops and trainings and provided technical assistance and continuous coaching and mentoring. Workshops and trainings (on message and material design, graphic design, and M&E) were provided to staff of all 3 units together to ensure a uniform understanding of SBCC and to facilitate learning and collaboration across the units. Knowledge management was integrated into many workshops, both to introduce the concept to participants and to demonstrate how it could support SBCC work. For example, an SBCC capacity strengthening training for 72 senior and junior health officers from 64 districts occurred in several increments from May to September 2013. As they received very little on-the-job training in this area, the 4.5-day curriculum covered how to design and implement strategic communication activities, how to use SBCC materials effectively, how to design and develop effective messages, monitoring and supervision for SBCC, as well as KM for SBCC.

As KM was a new concept for the health officers, the trainers first had to introduce the concept and ensure the participants understood it. After the introduction, the participants discussed typical challenges in public health, such as national guidelines not being updated or shared, program managers not being sure of which field workers were performing well, field workers leaving their jobs because they were not growing professionally, lack of opportunity to attend trainings, and SBCC materials being developed that have conflicting messages. Participants then worked in teams to discuss whether KM tools such as peer assist, mentorship, storytelling, online forums, and eLearning could help resolve any of these problems. The exercise helped participants understand how KM could support their activities and program goals.

Using a participatory assessment tool that scored various SBCC and KM capacity components from 1 to 4 (1 = poor, 4 = excellent), baseline and endline data were compared for each of the government units. For SBCC, in the IEM unit, the scores jumped from 1.92 to 3.42; for BHE, they increased from 1.97 to 2.64; and for IPHN, they increased slightly from 1.97 to 2.0. For KM, the IEM unit scores rose from 1.61 to 2.48; for BHE, they increased from 1.43 to 1.65; and for IPHN, they rose from 1.35 to 1.57. The IEM unit had the greatest improvement in SBCC and KM capacity compared with other units, and SBCC capacity scores improved more than KM scores overall. No statistical tests were conducted.

The endline post-assessments found that staff knowledge of how to design and implement SBCC activities had improved, and they increasingly used a strategic process for developing messages and materials. Staff also demonstrated greater ability to manage data, more appreciation for monitoring and evaluation, and improved leadership skills. However, BKMI staff did not successfully put KM processes in place. Although the units recognized the need, KM processes were not prioritized. Barriers included competing demands and limited information sharing. BKMI advocated improved KM with the line directors and at higher levels, including the Secretary of Health, specifically to develop processes and use tools that could help improve organizational effectiveness, knowledge sharing, and on-the-job skills. The MoHFW, however, had a greater interest in using external KM to improve coordination between SBCC stakeholders, rather than using it internally at the unit level.

Competing demands and limited information sharing impeded the use of KM processes.

### Lessons Learned

The BKMI project yielded many lessons that can inform future KM initiatives in the global health field.

#### Coordination of HPN SBCC Activities and Materials

Knowledge management in support of SBCC programs requires time and space to share knowledge in order to harmonize plans and align messages and activities within and across health sectors and among varying SBCC stakeholders. Coordination allows SBCC stakeholders opportunities to collaborate, share, and validate good practices and lessons learned, pool resources, avoid duplication of effort, and create and implement activities according to common quality standards. By establishing a systematic process for exchanging knowledge around SBCC programs and materials and institutionalizing a process through the working group and its members, BKMI sought to create a sustainable solution to chronic coordination issues. BKMI learned that to institutionalize effective coordination practices, it is important to take a multi-sector approach from the outset and to work closely with the MoHFW and align coordination activities with the Government of Bangladesh’s strategies and operational plans. In addition, coordination objectives and activities must be clear and inclusive of all who want to be involved. The BKMI team found that inclusiveness helped address common challenges from different angles and ensured that those implicated in SBCC activities in Bangladesh could have a voice and contribute to the national conversations occurring within the health system. Furthermore, for the effort to be sustainable, the MoHFW must take ownership of the group and drive the agenda.

#### Learning and Application of Integrated HPN SBCC Activities and Materials

The eHealth pilot presented an opportunity to use new technology to deliver effective HPN counseling to the community and improve knowledge in a standardized manner. While the BKMI team had concerns about using the netbooks with field workers, they quickly learned how to access the digital resources, demonstrating that technology can be a useful tool for facilitating SBCC and strengthening counseling skills. While not its original intention, field workers used the eLearning courses as counseling tools because clients found the courses entertaining and easy to understand. The BKMI team learned that providing field workers with digital resources on netbooks empowered them, increased their confidence on the job, and heightened their credibility in the community. The team also learned that the field workers would have benefitted from a more in-depth, in-person interpersonal communication and counseling training on how to use the netbook and its resources during counseling sessions. In addition, field workers would have been better supported if their supervisors had been part of the pilot and had been available to answer content-related questions and to help navigate the resources in the netbook to find those most appropriate for a particular counseling session. Finally, the project team learned that any activity involving technology requires ongoing IT and monitoring support.

Field workers used the eLearning courses as counseling tools—not by design but because clients found the courses entertaining and easy to understand.

The major activities of the BKMI project mutually reinforced each other. Capacity strengthening efforts through 3 units of the MoHFW contributed to improved SBCC capacity of staff and increased knowledge sharing through the coordination activities of the SBCC Working Group. All 3 units were involved in every stage of Toolkit and eLearning course development for the eHealth pilot that took place at the community level. The eHealth pilot was an important activity to link national coordination and community needs. Applying KM processes to SBCC activities, especially in the context of coordination, improved knowledge, helped people build on each other’s work, impacted efficiency, and ultimately strengthened the health system.

#### Strengthening KM and SBCC Capacity

Given the high staff turnover within the MoHFW, it is important to go beyond strengthening the capacity of individuals to focus on strengthening the capacity of the whole organization by setting up strong processes and employing state-of-the-art tools to ensure sustainability. Furthermore, organizations need to take responsibility for their own capacity strengthening initiatives by recognizing their importance and helping identify areas for improvement. Also, given competing time pressures, capacity strengthening plans should support and help meet the objectives and deliverables of operational plans. To be most effective, capacity strengthening efforts must include the full team of SBCC staff members to achieve a common level of SBCC ability throughout the organization. Integrating new technology as part of capacity strengthening can motivate staff and enable them to achieve deliverables more efficiently. Finally, the BKMI team learned that changing an organization’s culture and putting KM processes in place takes time and sustained advocacy.

With regards to sustainability of the BKMI activities, the project successfully advocated the institutionalization of the SBCC working group, and it continues to conduct SBCC and KM capacity building and coordination activities in the 3 units of the MoHFW. In addition, the project continues to update and expand the HPN Toolkit and 8 eLearning courses through a local organization called the Bangladesh Center for Communication Programs. The netbooks used for the eHealth pilot were deemed to not be scalable because of the cost associated with the netbook itself, as well as ongoing needs for IT and monitoring support. The netbooks loaded with the BKMI resources were therefore given to another USAID project in Bangladesh. Rather than pursuing scale-up through netbooks, the project is now exploring how it can scale-up use of the Toolkit and the eLearning courses to more health service providers in Bangladesh through the existing infrastructure, which includes mostly desktop computers in clinics and tablets among the health assistants.

## CONCLUSION

Many global health programs are grappling with issues related to coordination, learning and application, and capacity strengthening. Addressing these issues is critical across technical topics and sectors, as these foundational factors, if properly achieved, can increase efficiency, maximize resources, and contribute to both short- and long-term health goals.

The case study from Bangladesh illustrates how KM can improve coordination by creating and implementing a systematic process to exchange knowledge on a particular technical topic—in this case, SBCC programs and materials—as well as institutionalizing such a process to ensure sustainability. Toolkits increased HPN knowledge among field workers and eLearning courses strengthened field workers’ counseling and integrated messaging skills. These changes are hypothesized to improve service delivery quality. From a health systems strengthening perspective, this example illustrates that ensuring coordination among different actors, such as those working cross-sectorally in SBCC, supporting learning, and investing in capacity building, can contribute to improving health and development outcomes.

Further research would be beneficial in understanding the application of KM within the health sector. At a minimum, more rigorous studies that isolate KM activities and compare them to the absence of KM activities would provide stronger evidence of its effectiveness on health outcomes. Having a deeper understanding of how health care organizations use KM and further testing of various KM interventions within the health care context would be helpful in demonstrating the potential impact.^16^ Within the case of BKMI, we are in the process of evaluating impact data to more clearly make conclusions regarding the impact of strengthening coordination, capacity, and learning and application.

The discipline of KM as applied to global health and development would benefit from adopting more systematic processes, better defining the terms used to describe KM tools and processes, and critically examining the “how” to better integrate relevant theories into KM design, implementation, and research. Additionally, because the focus of today’s KM has shifted to KM practices for capturing knowledge that are fundamentally people-focused approaches, considering human and social factors in the KM puzzle is critical to potentially further the impact of health and development programs.

Because knowledge management has been informed by and used within disciplines outside public health, there is a crucial need to consider how to apply KM tools and processes from other fields to global health. Public health practitioners must recognize that one of the primary intangible assets we possess is knowledge and that we all require knowledge to solve the world’s pressing global health problems. The management of that knowledge is paramount but has yet to be viewed as such.

## Notes

**Peer Reviewed**
